# Use of Indocyanine Green Angiography for Real-Time Assessment of a Sternocleidomastoid Muscle Flap During Complex Facial Reconstruction

**DOI:** 10.7759/cureus.13970

**Published:** 2021-03-18

**Authors:** Lisandro Montorfano, Stephen J Bordes, Mauricio Sarmiento Cobos, Emmanuel Alejandro Garcia Lopez, Michael Medina

**Affiliations:** 1 General Surgery, Cleveland Clinic Florida, Weston, USA; 2 Surgical Anatomy, Tulane University School of Medicine, New Orleans, USA; 3 General Surgery, University of Miami Miller School of Medicine, Jackson Memorial Hospital, Miami, USA; 4 Otolaryngology-Head and Neck Surgery, Cleveland Clinic Florida, Weston, USA

**Keywords:** indocyanine green, icg angiography, sternocleidomastoid, muscle flap, facial reconstruction, scm muscle flap

## Abstract

Indocyanine green (ICG) angiography is a procedure that uses a fluorescent dye for a variety of medical diagnostics, including the real-time examination of blood flow in tissue. Herein, we report a case in which ICG angiography was used to assess the viability of a sternocleidomastoid (SCM) muscle flap during post-parotidectomy facial reconstruction. To our knowledge, this is the first report documenting the intraoperative use of ICG for the evaluation of SCM flap perfusion. ICG angiography may prove beneficial for cases involving complex reconstructions and suspected organ hypoperfusion.

## Introduction

Parotid gland neoplasms are the most common neoplasms of the salivary glands. Tumor resection may involve superficial, near-total, or total parotidectomy depending on the location and extension of the tumor. Patients are often left with a cosmetic deformity in the preauricular, infraauricular, and retromandibular regions [1,2]. Most parotidectomy reconstructions can be completed with local or regional flaps. Various grafts have been used to reconstruct the symmetry of these defects, such as superficial temporal fascial (STF) flaps, sternocleidomastoid (SCM) muscle flaps, superficial muscular aponeurotic system (SMAS) flap, microvascular flaps, and free fat grafts among others [3]. Various technologies providing an objective assessment of flap perfusion, such as indocyanine green (ICG) fluorescent angiography, have been developed [4-6]. We report the first case, to our knowledge, in which ICG angiography was used to assess perfusion and identify intraoperative ischemia in an SCM flap used for post-parotidectomy facial reconstruction.

## Case presentation

A 43-year-old Caucasian woman with pleomorphic adenoma of the parotid gland came to our hospital for a lateral parotidectomy and reconstruction. General anesthesia was administered, and the patient was intubated. Electrodes were placed for facial nerve monitoring along the frontalis, orbicularis oris, orbicularis oculi, and mentalis in addition to ground and reference electrodes. All electrodes were connected to the Medtronic nerve integrity monitor and proper functioning of the unit was confirmed. The patient’s face and neck were then prepped and draped in a sterile fashion. We performed a modified Blair incision with a cervical extension. This incision was performed with a 15-blade scalpel and carried down through the skin and subcutaneous tissue to the fascia of the parotid. Skin flaps were elevated just superficial to the parotid fascia with a Harmonic Focus device (Ethicon US, LLC, Cincinnati, OH). Elevation was carried out until the anterior border of the parotid gland was clearly defined. The skin flap was retracted with Lone Star hook retractors to maintain position throughout the procedure.

The attachment of the earlobe to the parotid fascia was carefully dissected, and we began to separate the parotid gland from the tragal cartilage by incising the attachments of the fascia with blunt and sharp dissection. The tragal pointer was identified. We released the attachments of the parotid gland to the sternocleidomastoid muscle and mobilized the gland anteriorly away from the mastoid tip and the sternocleidomastoid muscle. The posterior belly of the digastric was then identified.

Subsequently, we located a lymph node in the level II/infraparotid area. We carefully dissected this node and sent it to pathology for a frozen section, which confirmed a benign result. We then identified branches of the facial nerve, particularly the inferior division, and traced them posteriorly. We were able to dislocate the mass inferiorly as we dissected parotid tissue using a McCabe nerve dissector, Harmonic Focus device, and bipolar electrosurgery instrument. The parotid tumor was reflected away from and inferior to the nerve. The inferior branches were carefully mobilized at this point, and we completed the resection of the parotid tail with the tumor intact. The parotid gland was sent for a frozen section, which confirmed a benign tumor. The facial nerve was grossly intact and responded to minimal stimulation prior to skin closure. A facial defect of 3x2 cm was noted.

**Figure 1:**
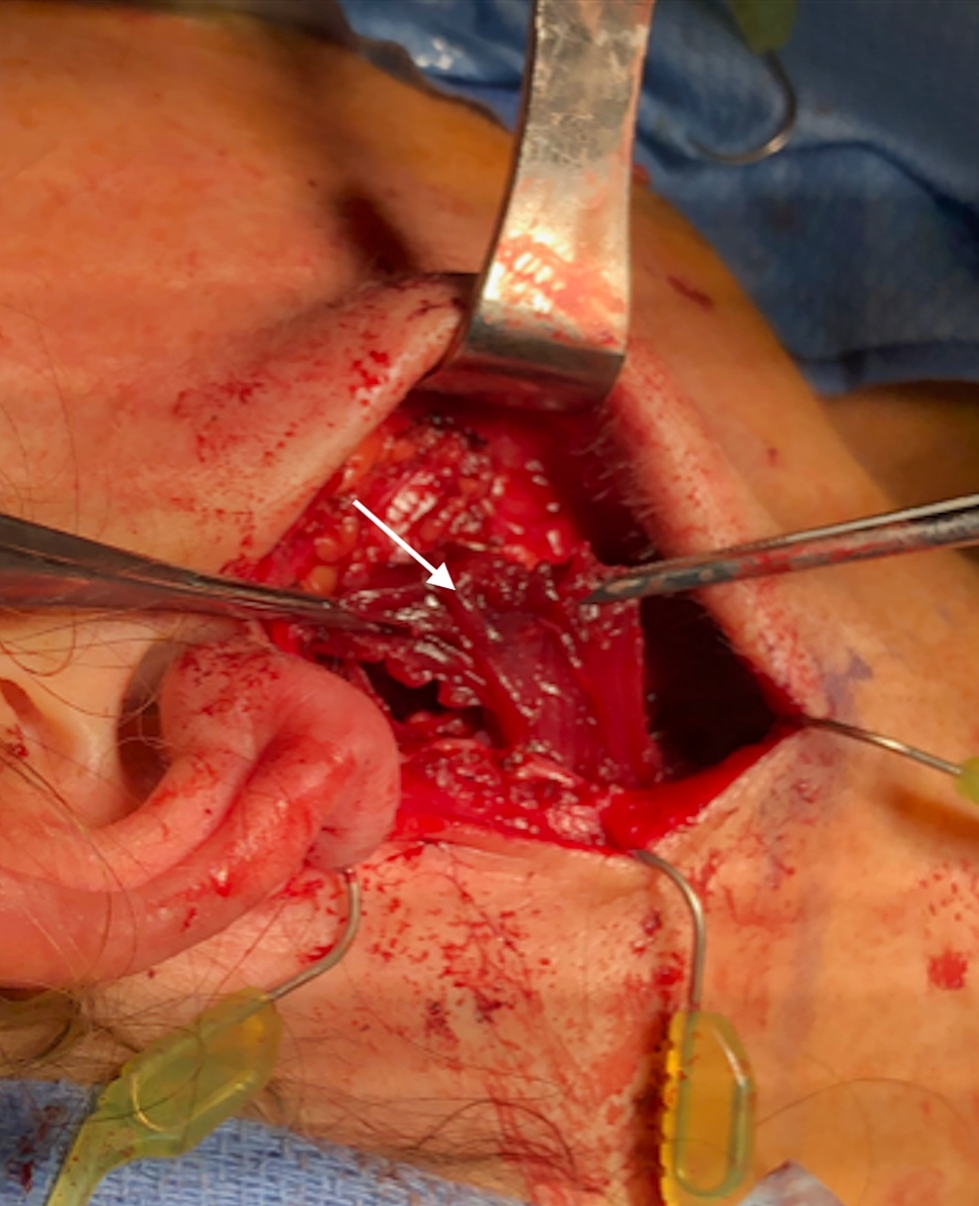
SCM flap mobilized with concerns of poor perfusion in the distal portion. SCM: sternocleidomastoid

**Figure 2:**
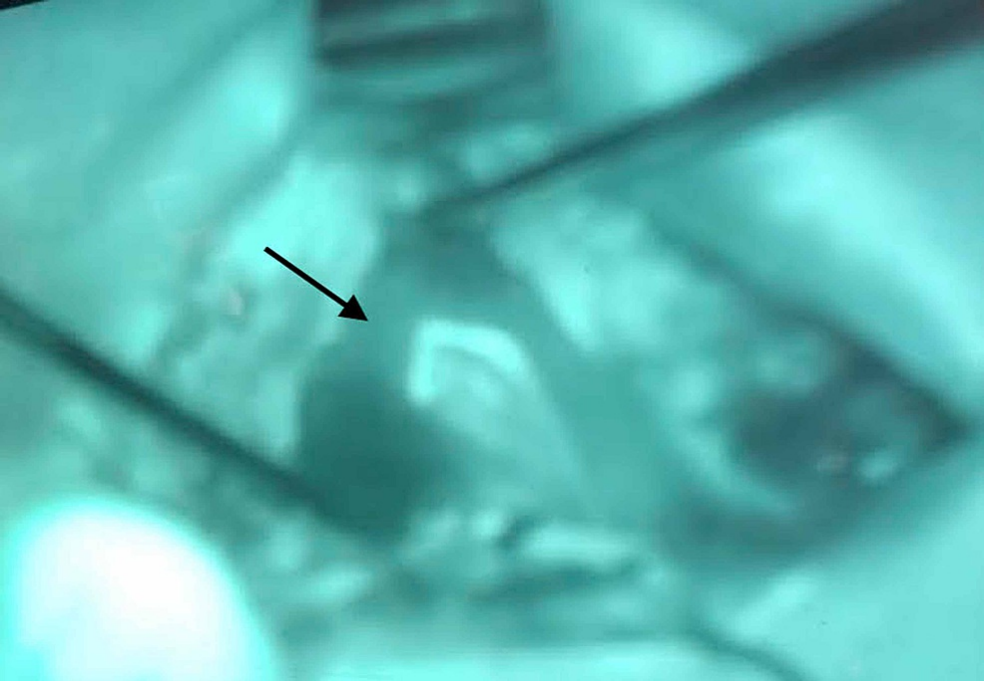
ICG perfusion showing poor perfusion of the distal portion of the SCM flap. ICG: indocyanine green; SCM: sternocleidomastoid

**Figure 3:**
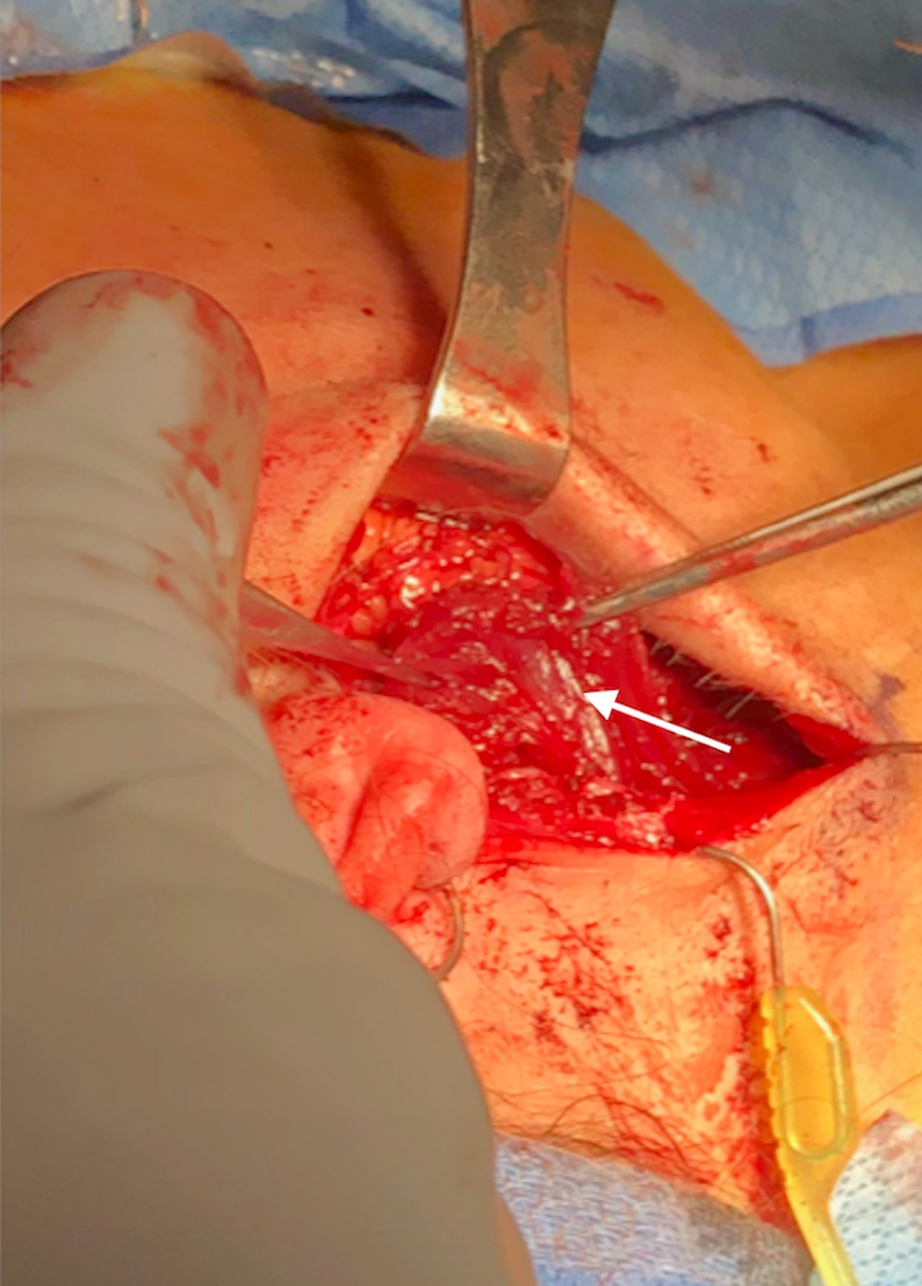
SCM flap following resection of the distal ischemic region. SCM: sternocleidomastoid

**Figure 4:**
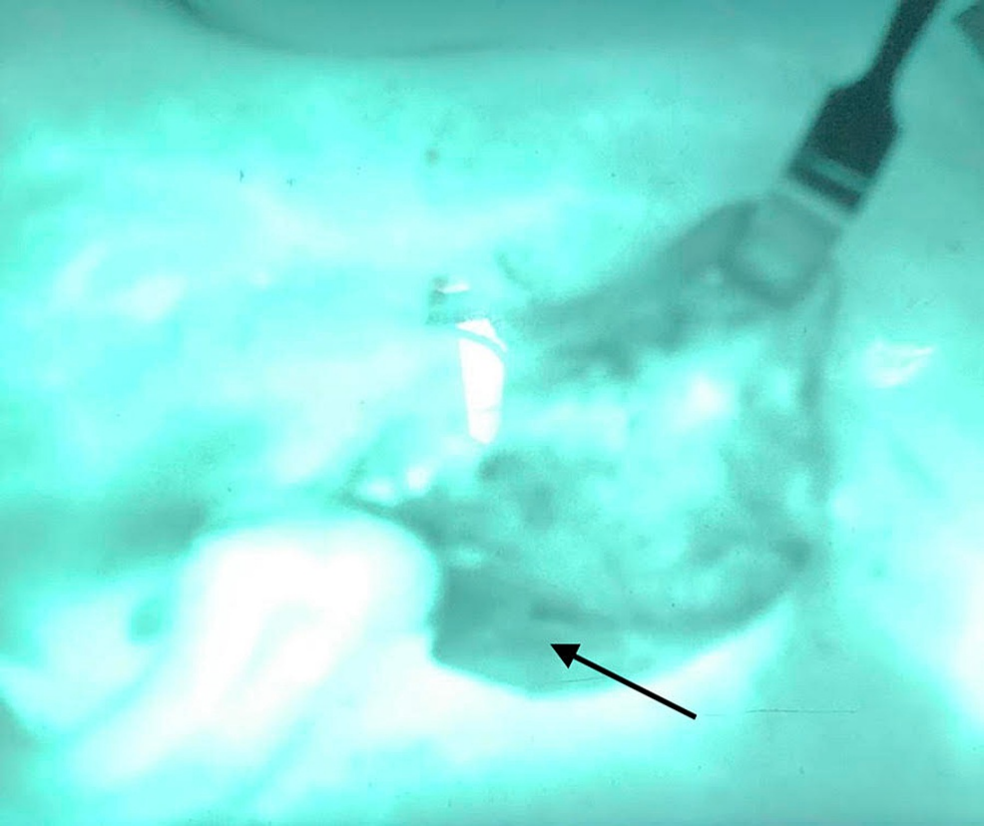
ICG perfusion showing the SCM flap sutured in place with good perfusion. ICG: indocyanine green; SCM: sternocleidomastoid

**Figure 5:**
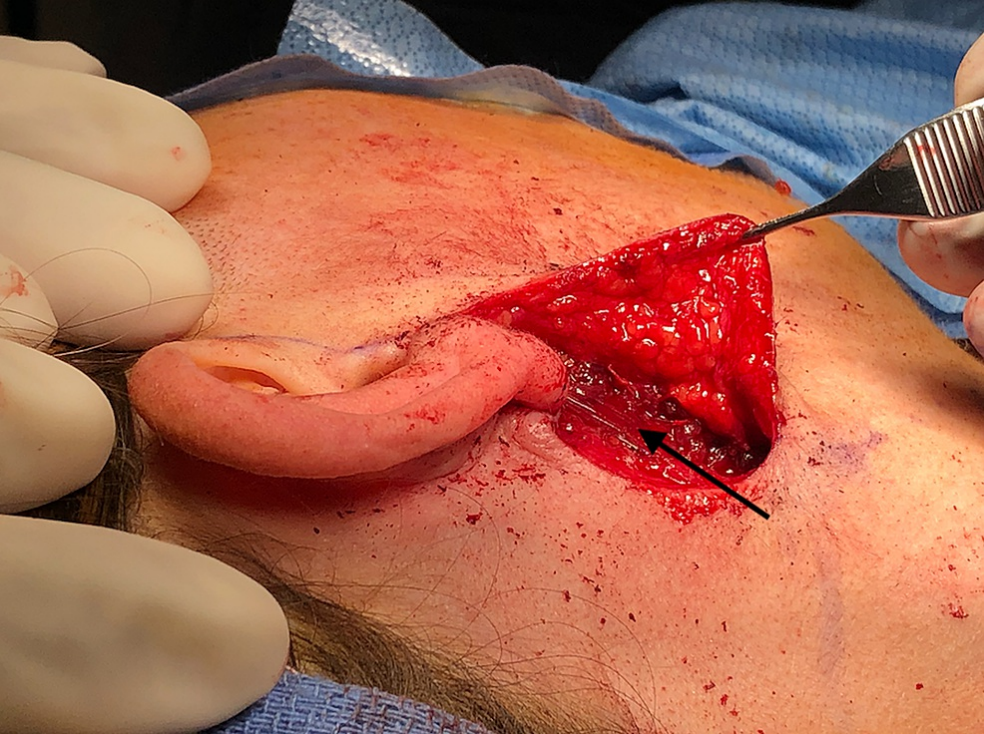
SCM flap sutured in place. SCM: sternocleidomastoid

A sternocleidomastoid flap was then used to facilitate reconstruction (Figure 1). We used indocyanine green angiography to assess the viability of the SCM flap. The distal part of the flap did not have appropriate perfusion (Figure 2), and this portion was subsequently trimmed (Figure 3). As a result, we dissected the base of the flap to gain more length. ICG angiography was used once again to check for viability. Sufficient perfusion was noted. The flap was then rotated anteriorly and superiorly, which improved the facial defect. The 3-0 Vicryl sutures secured the flap (Figures 4, 5). The surgical wound was rinsed with sterile saline solution and hemostasis was attained with bipolar cautery. We placed a #15 Jackson-Pratt drain and secured it to the skin with a 3-0 nylon suture. The incision was closed in layers using 3-0 and 4-0 Vicryl sutures for subcutaneous and deeper tissues while 4-0 and 5-0 nylon sutures were used for skin closure. 0.25% Marcaine was injected into the incision site. Antibiotic ointment was applied. The patient was awakened from general anesthesia and transferred to the recovery room in stable condition. She was seen in the clinic for follow-up two weeks postoperatively at which time no complications were noted.

## Discussion

Intravenous indocyanine green fluorescent dye consists of water-soluble tricarbocyanine molecules that emit infrared energy upon excitation by a light source. The emanating energy can be detected and visualized using a near-infrared light detector [7]. ICG has a half-life of 3.4 minutes and does not possess any toxic properties [8]. The molecules rapidly bind plasma lipoproteins and are excreted through the hepatic system via the first-pass effect [8]. In a large study, urticaria and anaphylactic reactions were found to occur at a rate of four of 240,000. Over the past 34 years, 17 adverse reactions have been documented that may be associated with the 5% sodium iodine used for solubility in ICG [9]. Overall, ICG is a safe diagnostic tool.

ICG was initially approved by the FDA in 1956 for evaluating cardiac output, ophthalmic vasculature, and hepatic blood flow [7,8,10]. Its use was subsequently expanded to determine the extent of oncological resections, evaluate intestinal anastomoses, predict parathyroid function following thyroidectomy, and assess perfusion in flaps [11]. The benefits of ICG are numerous such as its fast washout and high affinity to protein, which decrease risks of toxicity and leakage from intravascular space. Furthermore, multiple doses can be given until the ischemic areas are localized. This opens a possible area of research to assess the area necessary for excision when using specific muscle flaps.

Our case exemplifies an intraoperative application of ICG angiography for a sternocleidomastoid muscle flap for facial reconstruction. ICG angiography was used to assess the vascularization of an SCM muscle flap. To our knowledge, this case constitutes the first of such report in the literature. Initially, we observed poor vascularity in the distal portion of the flap and thus trimmed a portion of the muscle. Following the muscle resection, ICG angiography was again used to confirm adequate flap perfusion.

The SCM muscle is supplied by the occipital artery superiorly, superior thyroid artery mid-body, and transverse cervical artery inferiorly. SCM flaps work well for facial contour reconstruction following major excision as they have better outcomes overall and fewer complications such as Frey syndrome [12].

The benefits of ICG angiography have been seen in various contexts. In a recent retrospective study, the reliability of the supraclavicular artery flap and the thoracic branch of the supraclavicular artery (TBSA) flap was increased with the utilization of ICG angiography [13]. These flaps survived without necrosis and increased their length from a normal 30 cm to 35 cm with a thinner flap in certain cases [13]. A study of smokers and patients with comorbidities including obesity showed that ICG angiography decreased the risk of skin flap necrosis following mastectomy [14]. A retrospective study demonstrated the efficiency of harvesting superior gluteal artery perforator (SGAP) flaps with ICG angiography, which detected superior gluteal artery perforators for the flap design and thus ensured adequate vascularity [15]. These flaps survived beyond six months with no major complications [15]. A cadaveric study showed that ICG angiography allowed for the harvesting of a more favorable gracilis myofasciocutaneous flap, in which taking adjacent deep fascia increased the probability of a viable skin paddle [16].

With ICG angiography, hypoperfusion and ischemia can be addressed promptly with intraoperative debridement, which can prevent further surgical interventions, possible infections, delayed wound healing, and other complications [17,18]. As in our case, perfusion in the distal part of the flap can be unreliable, and the extent of the potentially ischemic flap in need of resection is unknown in the absence of techniques such as ICG angiography. ICG allows for intraoperative decision-making when determining flap viability [19]. As a result, intraoperative ICG for muscle flaps has the potential to decrease the length of individual hospital stays and thus associated economic costs. 

Moving forward, it will be important to systematize a more objective assessment of tissue perfusion in intraoperative settings, which may standardize the use of ICG angiography for muscle flaps and aid even the most experienced surgeons. Further cases are necessary to establish clinical significance.

## Conclusions

Based on our findings, the use of indocyanine green angiography for real-time assessment of intraoperative perfusion in sternocleidomastoid muscle flap reconstruction for post-parotidectomy facial defects has shown to be highly feasible. ICG angiography may be especially beneficial for cases in which complex reconstructions are performed and flap hypoperfusion is suspected.
